# Awareness of exercise importance, information sources, and adherence in predialysis chronic kidney disease in Japan: a web-based cross-sectional study

**DOI:** 10.1186/s12882-026-04850-z

**Published:** 2026-02-26

**Authors:** Yuhei Otobe, Connie M. Rhee

**Affiliations:** 1 Graduate School of Rehabilitation Science, Osaka Metropolitan University, 2-1-132, Morinomiya, Joto-ku, Osaka, 536-8525 Japan; 2 Division of Nephrology, David Geffen School of Medicine at the University of California Los Angeles, Los Angeles, California, USA; 3 Nephrology Section, Veterans Affairs Greater Los Angeles Healthcare System, Los Angeles, California, USA

**Keywords:** Chronic kidney disease, Exercise, Physical activity, Awareness, Adherence, Frailty

## Abstract

**Background:**

Physical activity (PA) and exercise are key in the non-pharmacological management of chronic kidney disease (CKD). However, patient awareness, information sources, and adherence to advice are not well-established. Herein, we quantified the awareness of the importance of PA and exercise, identified information sources, described receipt of advice and adherence, and explored factors associated with poor adherence.

**Methods:**

We conducted a web-based survey of adults with predialysis CKD in Japan. Inclusion criteria included being of age ≥50 years, physician-diagnosed CKD, no prior/active receipt of dialysis or transplant, ability to report recent serum creatinine or estimated glomerular filtration rates, and independence in activities of daily living. Questionnaires assessed awareness of importance (four-level scale), information sources, receipt of advice from healthcare professionals (yes/no), and adherence among recipients. Frailty was measured using the Kihon Checklist. Among recipients of exercise advice, correlates of poor adherence were examined using a modified Poisson regression with robust variance.

**Results:**

Of the 312 respondents, 285 met the inclusion criteria. Awareness of the importance of PA/exercise was very or moderately evident in 72.7% of respondents, and diet awareness was similarly evident in 85.3%. Physicians (70.1%) and online articles (33.3%) were the most common information sources; physical therapists were rarely cited (4.4%). Overall, 71.6% of respondents reported receiving PA/exercise advice: 12.3% almost completely adhered, 51.0% mostly adhered, 31.9% did not adhere adequately, and 4.9% were unsure how to follow the advice. Frailty was independently associated with poor adherence.

**Conclusions:**

In predialysis CKD, awareness of exercise lags behind that of diet, information sources are physician-centered, and adherence to advice is often suboptimal, particularly in patients with frailty. These findings reveal a gap in implementation and underscore the need to standardize structured clinical advice and monitoring, integrate exercise professionals into kidney care teams, and provide tailored support for frail patients.

**Supplementary Information:**

The online version contains supplementary material available at 10.1186/s12882-026-04850-z.

## Background

Physical activity (PA) and exercise are central components of non-pharmacological management of patients with chronic kidney disease (CKD). Historically, some nephrologists in Japan have been cautious about exercise because of concerns that exercise-induced increases in proteinuria might adversely affect kidney function [1]. However, recent evidence supports regular PA and exercise as safe and beneficial for patients with CKD, with salutary effects on kidney function, cardiovascular risk, physical and cognitive function, and health-related quality of life [2–8]. In light of this, clinical guidelines and statements worldwide now recommend PA and exercise for patients with CKD [9–12], reflecting a paradigm shift from historical exercise restrictions to active prescriptions.

Despite a robust evidence base, patient awareness of the importance of PA and exercise remains insufficient [13]. Although most nephrology healthcare providers consider PA counseling beneficial within their scope of practice, such advice is not routinely provided in clinical practice [14, 15], consequently limiting the opportunities for patients to learn about the importance of PA and exercise. Moreover, even when patients recognize its importance, the sources from which they obtain information, whether from healthcare professionals, family or peers, mass media, or digital resources, remain poorly defined. To effectively promote the role of PA and exercise in CKD care, it is essential to better understand patient awareness and their information sources.

In addition, data on the number of patients with CKD who reported having received exercise advice and the extent of their self-reported adherence are limited. Access to dedicated PA and exercise programs remains outside the standards of care for most kidney services globally [16].

In Japan, few facilities within the national health insurance system have been developed to provide exercise guidance for patients with predialysis CKD, and the involvement of exercise professionals, such as physical therapists, remains uncommon [17]. Nevertheless, clinicians and/or other healthcare professionals may still provide exercise advice as part of routine care even without specific reimbursement. However, the proportion of patients who report receiving such advice at the national level is unknown. Moreover, adherence is not routinely assessed among recipients who receive advice, and patient-level characteristics associated with low self-reported adherence remain poorly defined.

To address these gaps, we aimed to (1) quantify patients’ awareness of the importance of PA and exercise in CKD care and describe the sources from which they obtain such information; (2) describe the prevalence of self-reported receipt of PA/exercise advice from healthcare professionals and subsequent adherence; and (3) identify patient characteristics associated with poor adherence. The findings of this study have important implications for CKD care and will inform dissemination strategies to raise awareness of the importance of PA and exercise. Furthermore, this study supports the tailoring of exercise counseling and interventions for patients at risk of poor adherence.

## Methods

### Study population

This was a web-based cross-sectional study that recruited participants from the online agency’s registered panel. The agency distributed study invitations to panel members and administered a screening questionnaire. A total of 312 predialysis CKD participants completed an online survey in February 2024. The inclusion criteria were as follows: age ≥ 50 years, diagnosed with CKD by a physician, no prior or active receipt of dialysis or kidney transplantation, ability to provide estimated glomerular filtration rate (eGFR) or serum creatinine values from recent blood test results conducted during clinic visits, and ability to perform activities of daily living (ADL) independently (e.g., eating, grooming, walking, climbing stairs, and dressing). The reason for including patients age ≥ 50 years was to capture not only older adults, but also middle-aged patients, as vulnerability and psychosocial challenges can begin during this time frame. A previous study involving patients with predialysis CKD with a mean age of 52 years reported a 16% prevalence of frailty [18], supporting the relevance of this age group. Participants were excluded if they could not provide blood test results from the past year or if their eGFR was ≥ 90 mL/min/1.73 m^2^.

### Demographic and clinical characteristics

We collected data on demographic and clinical characteristics, including age, sex, body mass index, eGFR, comorbidities such as diabetes mellitus (DM) and cerebral/cardiovascular disease (CVD), education level (≤9 years, 10–12 years, or ≥ 13 years), and employment status (employed, never employed, or retired). All demographic and clinical characteristics were self-reported via the online questionnaire. Participants were instructed to refer to their most recent laboratory test results within the past year when entering kidney function values. When serum creatinine was provided, we calculated eGFR using an equation developed for Japanese subjects [19]. When eGFR was provided, we used the reported value.

CKD stages G2, G3a, G3b, G4, and G5 were defined as eGFR values of 60–89, 45–59, 30–44, 15–29, and <15 mL/min/1.73 m^2^, respectively [20]. Frailty status was assessed using the Kihon Checklist (KCL), a self-reported survey consisting of 25 yes/no questions related to instrumental ADLs, physical function, nutrition, oral function, homebound status, cognitive function, and depressive symptoms [21]. Based on a previous study [21], a KCL score of ≥ 8 points was considered as having frailty.

### Study questionnaire

Patients were administered a study questionnaire that was comprised of the following questions:

#### Awareness of the importance of PA and exercise in CKD care

Participants were asked, “How aware are you of the importance of PA and exercise in CKD care?” Responses included “Very aware”, “Moderately aware”, “Not very aware”, or “Not at all aware.” For comparison, the same question was also asked regarding dietary management.

#### Sources of information on the importance of PA and exercise

Participants who answered “Very aware” or “Moderately aware” were further asked, “Where did you obtain information about the importance of PA and exercise?” Multiple responses were allowed with the following options: physicians, nurses, pharmacists, dietitians, physical therapists, other healthcare professionals, care managers, friends or family, TV/radio, books/magazines, newspapers, online articles, and social media.

#### Experience of receiving exercise advice

Participants were asked, “Have you received exercise or PA advice from healthcare professionals?” with the response options of “Yes” or “No.”

#### Adherence to received exercise advice

Participants who answered “Yes” to the question on experience of receiving exercise advice were asked, “How closely do you follow the instructions you were given?” Response options were: “Almost completely adhere,” “Mostly adhere,” “Do not adhere adequately,” and “Unsure how to follow.” Poor adherence was defined as responding “Do not adhere adequately” or “Unsure how to follow.”

### Statistical analysis

Descriptive statistics were used to summarize the participant characteristics. Continuous variables were presented as means with standard deviations or medians with interquartile ranges, as appropriate, and categorical variables were presented as numbers and percentages.

Factors associated with poor adherence to exercise advice among participants who had received instructions were examined using modified Poisson regression with robust variance estimation to calculate prevalence ratios (PRs) and 95% confidence intervals (CIs). Poisson regression was selected since the prevalence of poor adherence exceeded 10%, the threshold above which the odds ratios from logistic regression may substantially overestimate the corresponding risk ratios [22, 23]. Both univariable and multivariable models were constructed. Variables included in the multivariable model were age, sex, eGFR, DM, CVD, education, employment status, and frailty status. All statistical analyses were conducted using Stata/SE version 19.0 for Mac (Stata Corp., College Station, TX, USA).

## Results

### Participant selection process and demographic/clinical characteristics

**Fig. 1:**
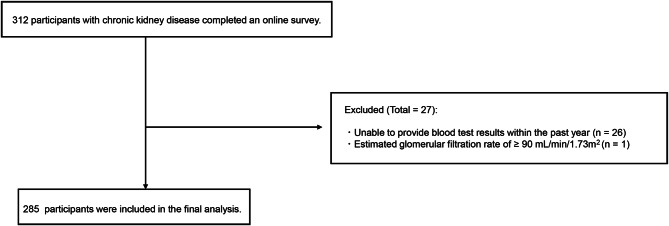
Flow diagram of the participant selection process

Of the 312 participants with CKD who completed the online survey, 27 were excluded: 26 were unable to provide blood test results from within the past year, and one had an eGFR ≥90 mL/min/1.73 m^2^. A total of 285 participants were included in the final analysis (Fig. 1). The demographic and clinical characteristics of the participants are presented in Table 1. The mean ± SD age of participants was 67.9 ± 7.8 years, and 249 (87.4%) were males. The mean ± SD eGFR was 40.7 ± 16.7 mL/min/1.73 m^2,^ and the distribution of CKD stages was as follows: G2, 34 (11.9%); G3a, 84 (29.4%); G3b, 91 (31.9%); G4, 54 (19.0%); and G5, 22 (7.7%). A total of 214 participants (75.1%) were classified as robust, while 71 (24.9%) were identified as frail.

**Table 1:** Demographic and clinical characteristics of participants

	Overall (*n* = 285)
Age, years (mean ± SD)	67.9 ± 7.8
Sex, male (n [%])	249 (87.4)
BMI, kg/m^2^ (mean ± SD)	23.3 ± 3.4
eGFR, mL/min/1.73 m^2^ (mean ± SD)	40.7 ± 16.7
CKD stage (n [%])	
G2	34 (11.9)
G3a	84 (29.4)
G3b	91 (31.9)
G4	54 (19.0)
G5	22 (7.7)
Comorbidities (n [%])	
Diabetes Mellitus	57 (20.0)
CVD	46 (16.1)
Education (n [%])	
≤9 years	4 (1.4)
10–12 years	82 (28.8)
≥13 years	199 (69.8)
Living alone (n [%])	24 (8.4)
Current employed (n [%])	129 (45.3)
KCL total score, points (median [IQR])	4.0 (2.0–7.0)
Frailty status (n [%])	
Robust	214 (75.1)
Frailty	71 (24.9)

SD, standard deviation; IQR, interquartile range; BMI, body mass index; eGFR, estimated glomerular filtration rate; CKD, chronic kidney disease; CVD, cerebral/cardiovascular disease; KCL, Kihon Checklist

### Awareness of PA and exercise in CKD care and sources of information

**Fig. 2:**
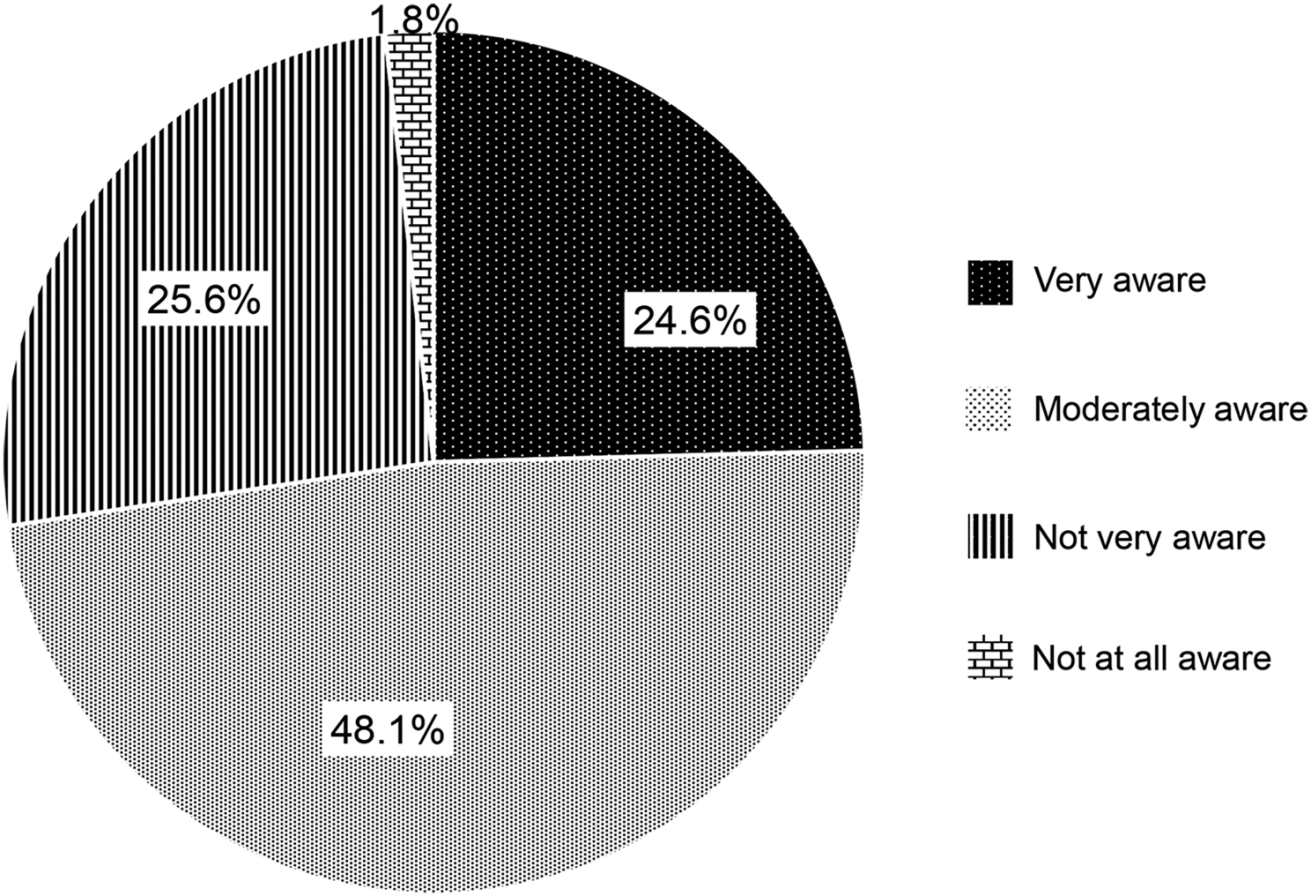
Awareness of the importance of physical activity and exercise in CKD care. Participants were asked, “how aware are you of the importance of physical activity and exercise in CKD care?”

The levels of awareness regarding the importance of PA and exercise in CKD care are outlined in Fig. 2. Seventy participants (24.6%) reported being “very aware,” 137 (48.1%) were “moderately aware,” 73 (25.6%) were “not very aware,” and five (1.8%) were “not at all aware.” Regarding dietary management, 118 (41.4%) reported being “very aware,” 125 (43.9%) were “moderately aware,” 37 (13.0%) were “not very aware,” and five (1.8%) were “not at all aware.”

**Fig. 3:**
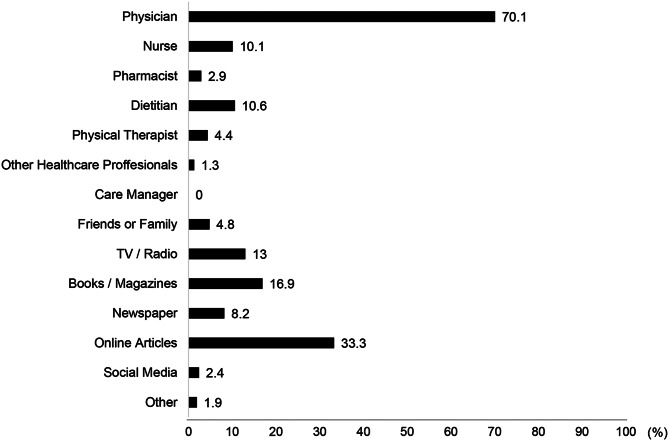
Sources of information on the importance of physical activity and exercise. Multiple answers were allowed for this question. Data are from participants who answered, “moderately aware” or “very aware” (*n* = 207) in response to the question, “how aware are you of the importance of physical activity and exercise in CKD care?”

The sources of information regarding the importance of PA and exercise are shown in Fig. 3. The most common sources were physicians (70.1%), online articles (33.3%), and books/magazines (16.9%), followed by TV/radio (13.0%), dietitians (10.6%), nurses (10.1%), newspaper (8.2%), friends/family (4.8%), physical therapists (4.4%), pharmacists (2.9%), social media (2.4%), other healthcare professionals (1.3%), and other sources (1.9%). Care managers were not reported as sources by any of the participants. Awareness of the importance of PA and exercise did not differ significantly by CKD stage (*p* = 0.31; Supplementary Table S1).

### Self-reported receipt of exercise advice and adherence level

**Fig. 4:**
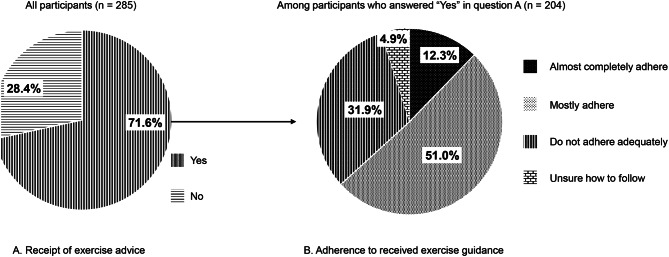
Self-reported receipt of exercise advice and adherence level. (**A**). Receipt of exercise advice. (**B**). Adherence to received exercise advice note: (**A**): all participants (*n* = 285) were asked, “have you received exercise or physical activity advice from healthcare professionals?” (**B**): among participants who reported receiving exercise advice (*n* = 204), we asked, “how closely do you follow the instructions you were given?”

Of the 285 participants, 204 (71.6%) reported having received PA or exercise advice from healthcare professionals, whereas 81 (28.4%) did not (Fig. 4A). Among those who had received advice (*n* = 204), 25 (12.3%) reported “almost complete adherence to the instructions,” 104 (51.0%) responded that they “mostly adhere,” 65 (31.9%) responded that they “do not adhere adequately,” and 10 (4.9%) were “unsure how to follow the instructions” (Fig. 4B). Receipt of PA and exercise advice differed by CKD stage (*p* = 0.015; Supplementary Table S1), with a higher proportion in later stages, whereas adherence level did not differ significantly by CKD stage (*p* = 0.67; Supplementary Table S2).

### Factors associated with poor adherence to exercise advice

In the modified Poisson regression analysis (Table 2), the multivariable model included age, sex, eGFR, DM, CVD, education, employment status, and frailty status. Frailty was significantly associated with poor adherence to exercise advice in both the univariable model (prevalence ratio [PR], 1.81; 95% confidence interval [CI], 1.28–2.55; *p* = 0.001) and the multivariable model (PR, 1.63; 95% CI, 1.14–2.32; *p* = 0.007). No other factors were significantly associated with poor patient adherence.

**Table 2:** Factors associated with poor adherence to exercise advice among participants reporting receipt of advice: a modified Poisson regression analysis

Factors	Reference	Category	Univariable			Multivariable		
			PR	95% CI	p	PR	95% CI	p
Sex	Male	Female	0.77	0.40–1.48	0.44	0.66	0.36–1.24	0.20
Age	per-1 year increase	–	0.98	0.95–1.00	0.07	0.97	0.95–1.00	0.05
eGFR	per-1 mL/min/1.73 m^2^ increase	–	0.99	0.99–1.01	0.81	1.00	0.99–1.01	0.83
Diabetes Mellitus	No	Yes	1.33	0.91–1.93	0.14	1.22	0.84–1.77	0.30
CVD	No	Yes	0.76	0.42–1.36	0.36	0.81	0.46–1.42	0.46
Education	≤9 years	10–12 years	1.32	0.26–6.75	0.74	1.45	0.36–5.89	0.60
		≥13 years	1.01	0.20–5.13	0.99	1.13	0.28–4.51	0.87
Current employed	No	Yes	1.03	0.72–1.48	0.86	0.91	0.61–1.34	0.63
Frailty	Robust	Frailty	1.81	1.28–2.55	0.001	1.63	1.14–2.32	0.007

PR, prevalence ratio; CI, confidence interval; eGFR, estimated glomerular filtration rate; CVD, cerebral/cardiovascular disease

In a sensitivity analysis replacing continuous eGFR with CKD stage categories, CKD stage was not associated with poor adherence, while frailty remained significantly associated (Supplementary Table S3).

Poor adherence (outcome) was defined as responding “Do not adhere adequately” or “Unsure how to follow” to the question, “How closely do you follow the instructions you were given?” among the participants who received advice on exercise or physical activity from healthcare professionals (*n* = 204). The multivariable model included all the variables listed in the table.

## Discussion

In this cross-sectional study of participants with predialysis CKD, we characterized patients’ awareness of the importance of PA and exercise, identified their information sources for these areas, and described the self-reported receipt of exercise advice from healthcare professionals and subsequent adherence. Three principal findings were identified in this study. First, awareness of the importance of PA/exercise was, overall moderate to high, although lower than awareness of dietary management. Second, physicians were the dominant information source (70.1%), with online articles also frequently cited (33.3%); physical therapists, as exercise professionals, were rarely cited (4.4%). Third, while 71.6% of participants reported receiving exercise advice from healthcare professionals, more than one-third of recipients of exercise advice reported poor adherence or uncertainty regarding how to follow instructions. Notably, frailty was independently associated with poor self-reported adherence.

Intervention studies and meta-analyses have shown that regular physical activity and exercise training improve exercise tolerance, cognitive function, and health-related quality of life in people with CKD [5–8]. These studies have also shown favorable effects on kidney function and cardiovascular risk factors [2, 3]. Accordingly, clinical practice guidelines and consensus statements now recommend promoting regular PA and exercise as part of CKD care [9–12]. Therefore, improving patients’ awareness of the importance of PA/exercise is a critical first step toward translating these recommendations into sustained behavior in routine practice.

Despite these recommendations, gaps remain in how patients perceive and act on PA/exercise in CKD care. In this study, we found that awareness of the importance of PA and exercise in CKD care was lower than awareness of dietary management. Moreover, information sources remain largely physician-centered, with limited involvement from other healthcare professionals. In our sample, 72.7% were “very/moderately” aware of PA and exercise versus 85.3% for diet. Physicians were the dominant source (70.1%), whereas physical therapists, as exercise professionals, were rarely cited (4.4%). Physician visits remain the primary channel for dissemination; however, outpatient time constraints and variability in exercise prescription knowledge (e.g., frequency, intensity, time, type, volume, and progression [FITT-VP] principles) may limit the specificity and consistency of messages delivered in routine care. Consistent with this, a survey of nephrologists reported that barriers to providing exercise advice included competing priorities, time constraints, limited knowledge of specific exercise prescriptions, and concerns that patients would not follow the advice of non-exercise experts [14]. Accordingly, greater involvement of exercise professionals, such as physical therapists, is warranted.

In practice, multidisciplinary care that includes physical therapists has been associated with a more favorable prognosis [24], and integrating exercise professionals into kidney care teams is recommended [25, 26]. However, in current practice, partly owing to reimbursement constraints, the participation of exercise professionals, such as physical therapists, in kidney care teams, remains limited [17, 27]. Collectively, policy actions to embed exercise professionals within kidney care teams are needed to enhance the dissemination of the importance of PA and exercise.

Online articles were identified as an information source by 33.3% of participants, second only to physicians. Online articles are increasingly recognized for their capacity to deliver PA/exercise information and support large populations in a potentially equitable and cost-effective manner [26, 28]. Accordingly, online articles are useful channels for raising awareness regarding the importance of PA and exercise. However, variability in information quality and readability, and the risk of misinformation and commercial bias remain a concern [29–32]; therefore, quality assurance and equity safeguards are essential. From an implementation standpoint, it is vital to signpost patients with easy-to-understand, professionally vetted online resources endorsed by academic societies and healthcare institutions (e.g., Kidney Beam [33] and My Kidneys and Me [34, 35]). Additional accommodations are needed for older adults, patients with limited health/digital literacy, and those without access to digital devices or the internet; hence, providing paper-based materials and other low-barrier formats may be essential. Accordingly, online resources should be leveraged while ensuring quality and equity to promote awareness of the importance of PA and exercise and support exercise advice.

Despite relatively high rates of exercise advice, adherence remained suboptimal, potentially indicating a gap between counseling and sustained behavior. In this study, 71.6% of participants reported receiving PA and exercise advice from healthcare professionals, yet among recipients of this advice, 36.8% reported poor adherence or were unsure how to follow the instructions. Several factors may contribute to this gap, including limited specificity and/or individualization of advice (e.g., lack of explicit FITT-VP parameters and written plans) [26, 36], insufficient follow-up [37], and outpatient time constraints [37]. Notably, the subgroup that was “unsure how to follow” suggested comprehension and implementation barriers rather than motivation alone, underscoring the need for clear, brief, and progressive prescriptions, safety guidance, and preference-aligned activities. Therefore, standardizing brief, structured exercise advice during clinic visits, with scheduled follow-ups, and referrals to exercise professionals (e.g., physical therapists), may help improve adherence and close the gap between counseling and sustained behavior change.

Frailty was associated with poor adherence to the exercise advice, necessitating targeted implementation efforts in this group. Symptoms and circumstances commonly observed in patients with frailty, including reduced exercise tolerance [38], fatigue [39], fear of falling [40], cognitive decline [41], psychological distress [42], and limited social support [43], can hinder the translation of understanding to the initiation and sustained practice of exercise. These features indicate that in-clinic advice, followed by unsupervised home exercises, may be insufficient for patients with frailty. Implementation should be personalized in both mode and setting and guided by the assessment of physical, cognitive, psychological, and social functions, including exercise-related risks [10, 44, 45]. When feasible, supervised or semi-supervised options should be offered through community resources (e.g., municipal classes, senior centers, and walking groups). Supplementing clinic-based advice with digital remote engagement and caregiver or peer support may be helpful for patients with frailty [46–48]. Accordingly, patients with frailty require individualized, supported pathways rather than advice alone to initiate and sustain exercise.

This study has several strengths. First, we sampled participants across almost all prefectures in Japan, reducing single-center bias. Second, by evaluating the awareness of PA and exercise alongside dietary management, we contextualized the relative awareness gap. Third, we quantified the information sources and distinguished advice receipt from adherence, thereby clarifying the translation gap between counselling and exercise adherence.

However, the significant limitations of this study warrant consideration. First, its cross-sectional design precluded causal inferences, including the directionality of the association between adherence to exercise advice and frailty. Second, the receipt of exercise advice and adherence were assessed by participant self-report; we did not capture the content or dose of counseling or objective measures of exercise behavior. In addition, demographic and clinical information, including comorbidities and kidney function values, were self-reported and not verified against medical records, which may have introduced measurement error. Frailty was assessed using the KCL, which has not been specifically validated in CKD populations, potentially leading to misclassification. Given these measurement constraints, residual confounding factors cannot be excluded. Future studies should document the counseling delivered and verify exercise implementation and functional status using objective measures. Third, using a web-based survey might have introduced sampling bias, as participants with higher digital literacy are likely overrepresented and may not reflect the broader CKD population. Moreover, the sample was predominantly male, further limiting generalizability.

## Conclusions

This web-based cross-sectional survey of participants with predialysis CKD revealed that the patients’ awareness of the importance of PA and exercise was moderate, lagging behind their awareness of diet. Information sources were mainly from physicians and online articles, whereas physical therapists were rarely cited. Most participants reported receiving exercise advice from healthcare professionals; however, adherence was often poor, and frailty was associated with non-adherence. These findings highlight an implementation gap. Providing standardized, structured clinical advice with scheduled follow-up, integrating exercise professionals into kidney care teams, and using high-quality online resources may help convert counseling into sustained exercise in CKD care. Particularly, patients with frailty are likely to struggle with adherence. Additional support and personalized delivery, including supervised community management, may be essential.

## Electronic supplementary material

Below is the link to the electronic supplementary material.

Supplementary Material 1

Supplementary Material 2

Supplementary Material 3

## Data Availability

The data used in the current study are not publicly available but can be obtained from the corresponding author upon reasonable request.
