# Supplementary material for: Awareness of exercise importance, information sources, and adherence in predialysis chronic kidney disease in Japan: a web-based cross-sectional study
Source: BMC Nephrol. 2026 Feb 26;27:211. doi: 10.1186/s12882-026-04850-z (PMC13041123; doi:10.1186/s12882-026-04850-z)
Supplement: Supplementary file 1 — Supplementary Material 1 [file 12882_2026_4850_MOESM1_ESM.docx]

Supplementary Table S1. Exercise awareness and receipt of PA/exercise advice by CKD stage among participants with predialysis CKD (n = 285).

|  | CKD stage | | | | |  |
| --- | --- | --- | --- | --- | --- | --- |
|  | G2  (n = 34) | G3a  (n = 84) | G3b  (n = 91) | G4  (n = 54) | G5  (n = 22) | *p* |
| **Awareness of PA/exercise importance, n (%)** |  |  |  |  |  | 0.31 |
| “Moderately aware” or “Very aware” | 22 (64.7) | 56 (66.7) | 70 (76.9) | 43 (79.6) | 16 (72.7) |  |
| “Not very aware” or “Not at all aware” | 12 (35.3) | 28 (33.3) | 21 (23.1) | 11 (20.4) | 6 (27.3) |  |
| **Self-reported receipt of exercise advice, n (%)** |  |  |  |  |  | 0.015 |
| Yes | 20 (58.8) | 54 (64.3) | 65 (71.4) | 47 (87.0) | 18 (81.8) |  |
| No | 14 (41.2) | 30 (35.7) | 26 (28.6) | 7 (13.0) | 4 (18.2) |  |
