# Supplementary material for: Awareness of exercise importance, information sources, and adherence in predialysis chronic kidney disease in Japan: a web-based cross-sectional study
Source: BMC Nephrol. 2026 Feb 26;27:211. doi: 10.1186/s12882-026-04850-z (PMC13041123; doi:10.1186/s12882-026-04850-z)
Supplement: Supplementary file 2 — Supplementary Material 2 [file 12882_2026_4850_MOESM2_ESM.docx]

Supplementary Table S2. Adherence level by CKD stage among participants who reported receiving exercise advice (n = 204).

|  | CKD stage | | | | |  |
| --- | --- | --- | --- | --- | --- | --- |
|  | G2  (n = 20) | G3a  (n = 54) | G3b  (n = 65) | G4  (n = 47) | G5  (n = 18) | *p* |
| **Adherence to exercise advice, n (%)** |  |  |  |  |  | 0.67 |
| “Almost completely adhere” or “Mostly adhere” | 12 (60.0) | 32 (59.3) | 46 (70.8) | 28 (59.6) | 11 (61.1) |  |
| “Do not adhere adequately” or “Unsure how to follow” | 8 (40.0) | 22 (40.7) | 19 (29.2) | 19 (40.4) | 7 (38.9) |  |
