# Supplementary material for: Awareness of exercise importance, information sources, and adherence in predialysis chronic kidney disease in Japan: a web-based cross-sectional study
Source: BMC Nephrol. 2026 Feb 26;27:211. doi: 10.1186/s12882-026-04850-z (PMC13041123; doi:10.1186/s12882-026-04850-z)
Supplement: Supplementary file 3 — Supplementary Material 3 [file 12882_2026_4850_MOESM3_ESM.docx]

Supplementary Table S3. Factors associated with poor adherence to exercise advice among participants reporting receipt of advice, using CKD stage categories in place of continuous eGFR.

| Factors | Reference | Category | Univariable | | |  | Multivariable | | |
| --- | --- | --- | --- | --- | --- | --- | --- | --- | --- |
|  |  |  | PR | 95% CI | *p* |  | PR | 95% CI | *p* |
| Sex | Male | Female | 0.77 | 0.40–1.48 | 0.44 |  | 0.65 | 0.36–1.17 | 0.15 |
| Age | per-1 year increase | – | 0.98 | 0.95–1.00 | 0.07 |  | 0.97 | 0.94–1.00 | 0.024 |
| CKD stage | G2 | G3a | 1.02 | 0.54–1.91 | 0.95 |  | 1.07 | 0.58–1.97 | 0.84 |
|  |  | G3b | 0.73 | 0.38–1.41 | 0.35 |  | 0.73 | 0.38–1.39 | 0.34 |
|  |  | G4 | 1.01 | 0.53–1.92 | 0.97 |  | 1.06 | 0.57–1.99 | 0.85 |
|  |  | G5 | 0.97 | 0.44–2.15 | 0.94 |  | 0.75 | 0.34–1.65 | 0.48 |
| Diabetes Mellitus | No | Yes | 1.33 | 0.91–1.93 | 0.14 |  | 1.22 | 0.84–1.79 | 0.30 |
| CVD | No | Yes | 0.76 | 0.42–1.36 | 0.36 |  | 0.83 | 0.48–1.44 | 0.50 |
| Education | ≤ 9 years | 10–12 years | 1.32 | 0.26–6.75 | 0.74 |  | 1.58 | 0.44–5.69 | 0.48 |
|  |  | ≥ 13 years | 1.01 | 0.20–5.13 | 0.99 |  | 1.20 | 0.34–4.20 | 0.78 |
| Current employed | No | Yes | 1.03 | 0.72–1.48 | 0.86 |  | 0.89 | 0.60–1.33 | 0.58 |
| Frailty | Non frailty | Frailty | 1.81 | 1.28–2.55 | 0.001 |  | 1.62 | 1.14–2.30 | 0.007 |

PR, prevalence ratio; CI, confidence interval; eGFR, estimated glomerular filtration rate; CVD, cerebral/cardiovascular disease.

Poor adherence (outcome) was defined as responding “Do not adhere adequately” or “Unsure how to follow” to the question, “How closely do you follow the instructions you were given?” among the participants who received advice on exercise or physical activity from healthcare professionals (n = 204). The multivariable model included all the variables listed in the table.
